# MODECP: A Multi-Objective Based Approach for Solving Distributed Controller Placement Problem in Software Defined Network

**DOI:** 10.3390/s22155475

**Published:** 2022-07-22

**Authors:** Chenxi Liao, Jia Chen, Kuo Guo, Shang Liu, Jing Chen, Deyun Gao

**Affiliations:** The School Electronic and Information Engineering, Beijing Jiaotong University, Beijing 100044, China; 20120060@bjtu.edu.cn (C.L.); 19111017@bjtu.edu.cn (K.G.); 21125049@bjtu.edu.cn (S.L.); 18111007@bjtu.edu.cn (J.C.); gaody@bjtu.edu.cn (D.G.)

**Keywords:** software-defined network, distributed controller placement, multi-objective, differential evolution algorithm

## Abstract

Software-Defined Network is an emerging networking paradigm that enables intelligent and flexible network management. Specifically, the design of the control plane is crucial. Therefore, in order to avoid a single point of failure, multiple controllers are deployed constantly in a distributed manner on the control plane. In this paper, we propose a controller placement approach based on multiple objectives (MODECP), including network delay, network security, load-balancing rate, and link occupancy. In the controller placement stage, an improved multi-objective differential evolution algorithm is proposed to search for controllers’ positions and assign switches to controllers reasonably. Furthermore, an improved affinity propagation algorithm is proposed to obtain the number of controllers placed in the network partition stage, comprehensively considering the delay, node security, and load. Simulations are performed based on several topologies from Internet Topology Zoo. Extensive results show that the proposed algorithm can realize trade-offs among multiple objectives and improve network performance in delay, security, controller load, and link occupancy compared to the single-objective based approach. Moreover, compared with the genetic algorithm and random placement algorithm, the proposed algorithm performs better with low latency, high security, low load rate, and low link overhead.

## 1. Introduction

With the continuous expansion of network service requirements and network scale, traditional network architecture has been unable to efficiently provide network resources for users, creating a bottleneck to improving performance. A software-defined network [1] separates the data plane from the control plane, which provides logically centralized and programmable control on the control plane to achieve intelligent management and optimization. Presently, the concept of SDN has been widely used in the fields of network function virtualization [2], the Internet of Things [3], mobile networks [4], and vehicle ad hoc networks [5], which helps to achieve intelligent resource management and service orchestration, information retrieval, distribution [6], etc. Consequently, designing a control plane for particular scenarios such as large-scale networks and software-defined satellite networking [7] has received extensive attention. In particular, the issue of controller placement is a typical problem. In SDN, there are one or more controllers to manage the network. At the same time, the control plane interacts with the data plane through southbound interface protocols such as OpenFlow [8] and P4Runtime [9]. The switch sends the data packets without forwarding rules in the flow table to the control plane. Next, the controller obtains the corresponding flow table rules and forwarding policies through calculation and sends them to the switch to realize network control.

However, in large-scale SDN or multi-domain SDN, a single controller may have two principal issues [10]: scalability and robustness. Therefore, the physically distributed controllers placement method is frequently used to solve the above problems. In other words, it is necessary to effectively place multiple controllers in the network to improve network performance and the efficiency of network management. Additionally, there are three architectures for distributed placement controllers [11]: flat, hierarchical, and hybrid deployments. In the flat controller design, each controller has its own local view and the global view of the entire network. In both cases, the controller communicates through east–west interface protocols. In the hierarchical controller design, the control plane adopts centralized management, and the top-level controller is set to manage the bottom-level controller. The bottom controller relies on the top controller for strategy calculation and forwarding of data packets. In the hybrid controller design, some simple control functions are transferred to the data plane so that the devices in the data plane participate in the control and decision processing.

Scalability in SDN [12] is usually manifested in the controller processing and response to flow requests, installing flow table rules, and cooperating with other controllers to manage the entire network. Specifically, Reference [13] evaluates the amount of traffic exchanged in the control plane between the switches and controllers and the number of flow rules installed, and it provides a quantitative model to quantify the maximum scalability of reactive network applications. By effectively placing multiple controllers, the single-point-of-failure problem can be avoided to enhance the control plane’s robustness and achieve traffic engineering, anomaly detection, fault restoration, resource orchestration, and other requirements. A key factor for the scalability of SDN in a large ISP (Internet Service Provider) is to study bandwidth and delay requirements for the control network to support the QoS requirements of the data plane [14].

In addition, security [15] is especially vital to a network. In order to avoid network paralysis caused by external malicious attacks or internal failures as much as possible, effective measures have to be taken to protect data from being leaked. The network security is enhanced by reasonably deploying the controller on nodes with high-security performance. To sum up, different multi-objective functions can be selected to optimize the placement of the controller according to the requirements of different network scenarios.

A reasonable controller placement model has to be established in the case of simultaneous optimization of multiple objectives. Two sub-problems should be solved:

(1) Determine the number of controllers.
(2) Determine the placement position of the controllers and which controller the switch should be assigned to.

Based on analysis of the problem, the controller placement problem is an NP-Hard problem [16], and the use of a heuristic algorithm is an effective method. 

Therefore, for the distributed controller placement of particular network scenarios, this paper proposes a heuristic-based multi-controller placement approach called MODECP. Firstly, multiple objective models are established, including the network end-to-end delay, network security, controller load rate, and link cost. In particular, the network end-to-end delay is expressed as switch–controller propagation delay, inter-controller propagation delay, processing delay, and transmission delay. The network security is modeled as the failure cost of the controller based on degree centrality, and the failure cost of the control link is based on betweenness centrality. Meanwhile, the link cost is modeled as the link bandwidth occupied by switch–controller communication and inter-controller synchronization. Secondly, an improved approach based on affinity propagation algorithm is proposed, which adaptively obtains a reasonable and effective number of controllers. Finally, we propose an improved multi-objective differential evolution algorithm that solves the controller placement problem and switches assignment to improve network performance efficiently. The main contributions of this paper are as follows:

(1) The controller placement problem is effectively solved, and the network delay, network security, controller load rate, and link cost are modeled. At the same time, a multi-objective model to solve the controller placement problem is obtained by weighing multiple objectives.
(2) On the one hand, an improved affinity propagation algorithm is proposed, which integrates the characteristics of network nodes, including delay, switch security, and load. On the other hand, a controller placement approach based on a multi-objective differential evolution algorithm is proposed to optimize performance objectives such as delay, security, load balancing, and link cost.
(3) The proposed method is simulated extensively. We compare it with various single-objective based schemes. In addition, we compare it to the multi-objective based genetic algorithm and the delay-based random placement algorithm. Furthermore, we compare the proposed approach under different topologies. The feasibility and efficiency of MODECP are verified by comparing the performance. In addition, to better evaluate the impact of delay and link state changes on the deployment results, we change the delay calculation parameters and the link failure states predicted using the SVM model to represent the performance of MODECP.

The structure of this paper is as follows. Section 2 briefly discusses related work, and Section 3 describes the system model and problem formulation of distributed controller placement. Section 4 proposes a MODECP approach based on the improved affinity propagation algorithm and multi-objective differential evolution algorithm. The proposed algorithm is simulated, and the performance is analyzed and evaluated in Section 5. Section 6 summarizes this paper.

## 2. Related Works

There are many strategies for the controller placement problem (CPP). In this section, optimization objectives and optimization algorithms are introduced for the controller placement.

### 2.1. Optimization Objectives for Controller Deployment

Network performance optimization parameters considered in existing solutions usually include reliability, load balancing, delay, and event response time. In order to reasonably place the controller and allocate the switch–controller mapping relationship, optimization is generally achieved by optimizing single or multiple network performance parameters. The algorithm proposed in [17] partitions the network to minimize the maximum delay between switches. Reference [18] considers the controller load factor, which is regarded as a k-center problem with capacity, significantly increasing the capacity of the control plane. Reference [19] defines the controller placement problem for load balancing, which minimizes the average delay between switches and controllers while maintaining load balancing among controllers. Some methods consider multiple optimization objectives. Reference [20] employs a bargaining game to search for the optimal placement strategy of the controller for minimizing the objectives of delay, communication overhead, and load balancing. In order to ensure that the communication between the switch and controller is not interrupted to guarantee the network’s reliability, Reference [21] proposes two reliable placement strategies. One is that there must be two disjoint control paths between the switch and the controller. The other considers that the switch must be connected to two different controller duplicates for efficient failover. In the multi-objective approach, some objectives are conflicting; for example, if the average delay in the switch–controller interaction is minimized, the average delay of the inter-controller becomes more extensive, which leads to the degradation of QoS. In addition, some solutions to the controller placement problem also consider other performance metrics, such as energy efficiency. Reference [22] proposes an energy-aware solution called GreCo, which aims to minimize network energy consumption. 

In the early period of network establishment, the secure placement of controllers is a challenging problem. Reference [23] proposes a controller placement strategy for multi-link failures with maximum resource utilization and minimum worst-case delay to achieve efficient network performance. Reference [24] proposes a heuristic algorithm to solve CPP with a single-link failure. In addition, the authors also exploit a greedy algorithm based on Monte Carlo simulation to solve CPP with multiple-link failures. Apart from this, the security of the network depends on the performance of the intrusion detection algorithm. Reference [25] proposes a machine-learning-based anomaly detection algorithm to quickly guide traffic classification and attack flow redirection problems to analyze unknown threats and attacks better.

### 2.2. Optimization Algorithms for Controller Deployment

From the perspective of optimization algorithms for solving controller placement problems, the common optimization methods for controller placement problems are based on integer linear programming (ILP), heuristics, clustering, etc. Reference [26] adopts hierarchical clustering to minimize the number of controllers while reducing the load difference between different controllers. Reference [27] proposes a network state-based traversal algorithm and a greedy algorithm that places the controller in polynomial time and improves the performance in the event of link failure. Reference [28] proposes a multi-objective genetic-algorithm-based controller placement strategy to minimize inter-controller delay and load distribution. In order to avoid repeated management and intervention of the controller and a sharp increase in link disconnection, Reference [29] considers the reliability and capacity of the controller, which determined the controller failure in advance. The authors propose a simulated annealing heuristic to solve problems on large-scale networks efficiently.

Furthermore, controller dynamic placement is also a challenge. Most of the existing controller placement methods consider static placement. However, since the traffic constantly changes in large-scale or multi-domain networks, it is necessary to place controllers dynamically. Currently, machine learning is commonly used in dynamic controller deployment. Reference [30] proposes a Deep Q-Network-based Dynamic Clustering and Placement (DDCP) approach, which significantly improves the network performance regarding response time and resource utilization. Reference [31] proposes a Deep Q-Network (DQN)-empowered dynamic-flow data-driven approach to adapt to the dynamic network environment with flow fluctuations. In Reference [32], an algorithm based on a quadratic program is designed to solve the controller placement problem, and a dynamic switch migration algorithm is developed to deal with network congestion.

In addition, Reference [33] quantitatively evaluated the control flow of ONOS, Floodlight, and POX controllers and obtained the control channel usage of the controller. The analysis of the control flow shows that the control flow is crucial to the scalability of the control channel, which also provides strong support for the necessity of establishing the delay model and link cost model. At the same time, the above research proves that the distribution of load in the network and the security composed of link failure and controller failure are of great significance to improving the network performance, which provides support for the establishment of the load model and security model to solve the problem of controller deployment.

In summary, Table 1 summarizes relevant studies that mainly optimize one or more objectives such as delay, load, reliability, and energy efficiency. At the same time, through the summary of existing relevant research, it is found that most of the studies have ignored the determination of the number of controllers. References [20,26,28] consider the number of controllers that need to be deployed in the network. However, these studies did not consider the characteristics of each node when determining the number of controllers. Thus, MODECP is proposed to solve the controller placement problem. Firstly, the number of controllers is adaptively generated in the network according to the node characteristics. Then, a multi-objective model is established to balance the multiple objectives simultaneously in the network, including end-to-end delay, network security, controller load, and link cost, to obtain approximately optimal positions for controllers and approximately optimal assignments for switches. 

## 3. System Model and Problem Formulation

In this section, we present the system model, including delay model, security model, controller load model, and link cost model. The symbols used in this paper are summarized in Table 2 for clarity.

### 3.1. System Model

#### 3.1.1. Network Model

We define the underlying network as an undirected graph composed of $G\left(N,E\right)$, where N represents the set of SDN switches and E represents the set of physical links. The number of controllers that will be placed on the network is defined as k, and the set of controllers is defined as $C=\{c_{1},c_{2},\ldots ,c_{k}\}$. Each controller manages a certain number of switches. Importantly, each controller is located on a switch in the network. That is, some nodes are responsible for both control and forwarding functions.

Moreover, two communication models exist for southbound interfaces in SDN, in-band and out-of-band [34]. In this paper, we adopt the switch-to-controller communication model with in-band connections. In a real network scenario, the servers are more widely distributed geographically, resulting in a higher cost of building the network. For the out-of-band communication model, it is necessary to add dedicated links for controlling traffic transmission and available physical ports for adding dedicated links to achieve network scalability. As a result, to reduce the cost of network deployment and efficiently utilize resources such as links and ports, it is more reasonable to adopt the in-band communication model between switches and controllers for practical network scenarios. Similarly, this paper adopts in-band communication between controllers. A competitive relationship exists between the switch–controller delay and the inter-controller delay. When reducing the switch–controller latency, the controllers are deployed relatively far from each other, increasing the inter-controller delay. Conversely, controllers are deployed relatively close together to reduce inter-control communication latency, resulting in an increase in switch–controller latency. If the controllers are required to be deployed geographically close to each other, then it is more reasonable to use out-of-band link interconnections between controllers. However, considering the actual network environment, the switch–controller delay is as significant as the inter-controller latency. Thus, controllers adopt in-band communication to balance switch–controller delay with inter-controller delay.

Furthermore, the controllers can be arranged through a flat or hierarchical structure. A hierarchical structure consists of multiple local controllers and a logically centralized root controller [35]. In this case, the local controllers handle frequently occurring events, while the root controller handles rarely occurring events. The controllers communicate with each other based on events and asynchronously. Meanwhile, the root controller employs a subscription-based approach to communicate with the local controllers. In a flat architecture, strict consistency algorithms such as the Raft algorithm are often used between controllers to maintain data consistency for all controllers to have a global view [36]. At the same time, controller-synchronized traffic shares the data plane links with the traffic of controller-managed switches. Therefore, updating forwarding rules between controllers at regular intervals is essential for efficient packet processing.

We assume that the capacity of each controller is the same as its packet processing rate, which is defined as Ca. The flow request rate of switch *i* is defined as $\lambda_{i}$, and each switch can be managed by only one controller. Since the traffic in the network is changing dynamically, we assume that the time interval of synchronization between controllers is $p_{sync}$. At the same time, we assume that the communication frequency between switches and controllers is disregarded.

#### 3.1.2. Delay Model

In this paper, the delay is crucial for improving network performance. Specifically, the delayed response of the network depends on the switch–controller interaction and the inter-controller timing synchronization that updates the global view, the packet transmission delay, and the processing delay.

The switch–controller interaction is represented as the average propagation delay between switch *i* and controller *j*, which is calculated by Equation (1) through the shortest path length between them. In addition, the physical link length of the network is calculated by the geographical latitude and longitude positions of the nodes to combine with the actual environment. The shortest path length is the combination of the number of hops of the shortest path and the length of the single-hop physical link.

(1) $$D_{ncavg}=\frac{1}{\left|N\right|\cdot v}\sum_{n_{i}\in N}\sum_{c_{j}\in C}d_{ij}^{nc}x_{ij},$$

Here, $d_{ij}^{nc}$ represents the shortest transmission path length between switch *i* and controller *j*, and $v=2\times 10^{8}\;m/s$ represents the propagation rate of electrical signals in cables. 

The inter-controller timing update forwarding rule is expressed as the propagation delay of inter-controller synchronization. Similarly, the delay of synchronization is calculated by the shortest path length between controllers, expressed as Equation (2):

(2) $$D_{ccavg}=\frac{\sum_{c_{i},c_{j}\in C}d_{ij}^{cc}}{v},$$

where $d_{ij}^{cc}$ represents the shortest transmission path length between controller *i* and controller *j*, and $v=2\times 10^{8}\;m/s$ represents the propagation rate of electrical signals in cables.

In considering a real network scenario, the time for network traffic to be sent from the switch or controller is defined as the transmission delay, which is calculated by Equation (3). Each controller processes the packets received from the switch, and this phase is accompanied by processing delays related to the hardware devices. The packet processing in the controller is modeled by the M/M/1 queuing model [37], which is computed through Equation (4).

(3) $$D_{trans}=\frac{p_{send}}{B_{l}},$$

(4) $$D_{proc}=\frac{1}{k}\sum_{c_{j}\in C}\frac{1}{Ca-\sum_{s_{i}\in N,c_{j}\in C}\lambda_{i}x_{ij}},$$

Here, $p_{send}$ is the packet size sent by the switch or controller, Ca is the average rate at which the controller processes packets, and $\lambda_{i}$ is the average flow request rate for switch *i*.

Therefore, in SDN, the delayed response of the network is defined as the end-to-end delay, which is calculated by the round-trip time of the packets in the network [34,36], represented by the following formula:

(5) $$D=2D_{trans}+D_{proc}+2D_{ncavg}+2D_{ccavg},$$

#### 3.1.3. Security Model

In any network topology, the degree centrality of a node [38] is the most direct measurement parameter to describe the centrality of a node in SDN. Specifically, the greater the degree of a node, the higher the degree centrality of the node, and the more influential the node is in the network. During network operation, the failure cost of each switch is assigned a different value due to its relative position and degree of influence on the network. Therefore, we define the failure cost of a switch as the degree centrality of a node. As the connectivity of the switch is greater, its relative position is more important in the network. At the same time, if it fails, it will significantly impact the network, so its failure cost will be greater. The failure cost of the switch is calculated by Equation (6):

(6) $$\varphi_{c}=\frac{\sigma_{c}}{\sum_{n\in N}\sigma_{n}},$$

where $\sigma_{c}$ is the degree centrality of controller *c*, and $\sigma_{n}$ is the degree centrality of switch *n* in the network.

In addition, the betweenness centrality of a link [39] measures how important a link is in the network. Link betweenness is defined as the ratio of the number of paths traversing a link to the total number of shortest paths among all the shortest paths. Specifically, a larger link betweenness means that the role of the link in the network is more critical. Therefore, we define the control link’s failure cost as the link’s betweenness centrality. If a link fails, the greater the link betweenness, the greater the impact on the network. We expect the set of control paths to contain paths with smaller link betweenness. The failure cost of the control path is calculated by Equation (7):

(7) $$\varphi_{e_{ij}}=\sum_{i,j\in N}\frac{\sigma_{ij}(e)}{\sigma_{ij}},$$

where $\varphi_{e_{ij}}$ is the edge betweenness of link *e* from switch *i* to switch *j*, $\sigma_{ij}$ is the total number of shortest paths from switch *i* to switch *j*, and $\sigma_{ij}(e)$ is the number of the shortest paths passing through link *e*.

When the controllers in the SDN adopt the distributed placement method, to ensure all the controllers obtain the same topology information on the whole network, one controller communicates with other controllers regularly. In this paper, we assume that nodes in the network will encounter malicious attacks or hardware and software failures, which cannot be recovered autonomously. In other words, if one or more controllers are attacked from outside, there is a certain probability that the control node or the control link will fail, resulting in information leakage and data loss.

Therefore, we expect to enhance the network’s security as much as possible to minimize the probability of data loss. The expected percentage of control node failures and the expected percentage of control link interruption are considered as the parameters of security, which are defined by the following formula:

(8) $$S=\frac{1}{1-\prod_{e_{ij}\in P_{ij},c\in C}(1-\varphi_{c})(1-\varphi_{e_{ij}})},$$

where S is the security of the network, and $P_{ij}$ is the set of shortest control paths between switch *i* and controller *j.*

To characterize the impact of link failures on controller deployment results, this paper simulates multiple-link failures to illustrate the impact of reduced security performance on network delay, load, and link overhead. To this end, we employ Support Vector Machine (SVM) [40], a supervised learning method, for predicting link failures. Each link and its state constitute a binary group $\left(e_{ij},s_{ij}\right)$. When $s_{ij}=1$, link $e_{ij}$ is not in failure. When $s_{ij}=0$, link $e_{ij}$ is in a disrupted state. We adopt the historical data of link states for link failure prediction. The prediction of link states is a nonlinear binary problem. Thus, the SVM model uses a radial basis function kernel, as in Equation (9):

(9) $$K(e_{ij},e_{ij}^{\prime })=exp(-\mu {\left\|e_{ij}-e_{ij}^{\prime }\right\|}^{2}),$$

where μ is the free parameter that controls the variance of the model.

#### 3.1.4. Load Model

The ideal load-balancing state in a network is one in which equal load is assigned to all controllers. In this section, we consider the average flow request volume of switches within a controller’s management scope to be the controller’s load. Through the allocation of switches, the load difference among all controllers is defined as the load deviation rate, denoted as *L*. The smaller the index, the better the network load-balancing performance after correctly placing controllers. The load deviation rate of the controller is calculated by Equation (10):

(10) $$L=\sqrt{\frac{1}{k}\sum_{\forall c\in C}{\left(l_{c}-l_{opt}\right)}^{2}},$$

where $l_{c}$ is the actual load of the controller *c*, and $l_{opt}=\lambda_{i}N/k$ is the load of the controller in the ideal state.

#### 3.1.5. Link Cost Model

To satisfy the growing number of service requirements and improve service quality in SDN, we expect to improve the scalability of SDN with minimal network congestion cost. In this paper, the network congestion cost is expressed as the link bandwidth consumption of each control path during network operation. Its control path includes control paths for switch–controller communication and inter-controller synchronization. Due to the limited network resources, we expect to reduce the link consumption of each control path, to minimize the link occupancy rate of the control path as much as possible and meet the user’s available bandwidth requirements. Each control link is included in the set of all control links, which is denoted as $P_{ij}$. The switch–controller link occupancy rate is calculated by Equation (11). The inter-controller link occupancy rate is calculated by Equation (12).

(11) $$B_{prop}=\sum_{e_{ij}\in P_{ij}}^{}\sum_{n_{i}\in N}^{}\sum_{c_{j}\in C}^{}\frac{z_{e_{ij}}\rho_{ne}x_{ij}}{B_{e}},$$

Here, $z_{e_{ij}}$ is a binary variable, indicating whether the link $e_{ij}$ belongs to the set of control paths. $\rho_{ne}$ is the link bandwidth consumption occupied by each switch–controller communication.

(12) $$B_{sync}=\sum_{e_{ij}\in P_{ij}}^{}\sum_{c_{i},c_{j}\in C}^{}\frac{z_{e_{ij}}p_{sync}\rho_{ce}}{B_{e}},$$

Here, $z_{e_{ij}}$ is a binary variable, indicating whether the link $e_{ij}$ belongs to the set of control paths. $p_{sync}$ is the frequency of timing synchronization between controllers. $\rho_{ce}$ is the link bandwidth consumption occupied by each inter-controller communication. 

Therefore, the link cost metric is defined as the utilization of all control path links of the whole network, which is calculated by the following formula:

(13) $$B=B_{prop}\times B_{sync},$$

### 3.2. Constraints

Next, we describe the constraints of the problem.

For a given network topology, the number of controllers to place is not known in advance. Therefore, when it is determined that the number of controllers to be placed is *k*, *k* switches should be selected as control nodes in the network. Constraint (14) ensures that *k* controllers are placed in the entire network when solving the controller placement problem.

(14) $$\sum_{c=1}^{N}y_{c}=k,$$

Constraint (15) ensures that each switch is managed by only one controller.

(15) $$\sum_{c=1}^{C}x_{nc}=1,\forall n\in N,\;x_{nc}\leq y_{c}$$

The processing capability of the controller in the real network environment is affected by the performance of its software and hardware. Therefore, to simulate reality, the amount of flow requests handled by the controller to the switch does not exceed the capacity of the controller, which is defined by Constraint (16).

(16) $$l_{c}=\sum_{n_{i}\in A_{c}}\lambda_{i}<Ca,\;\forall n_{i}\in A_{c},\forall c\in C$$

Link bandwidth is limited in the network. Constraint (17) ensures that the link consumption through which the switch–controller communication and the inter-controller synchronization pass does not exceed the bandwidth threshold of the link itself.

(17) $$z_{e_{ij}}\rho_{ne}\leq B_{e},\;z_{e_{ij}}\rho_{ce}\leq B_{e},\;\forall n\in N,\forall c\in C,\forall e_{ij}\in P_{ij}$$

Constraints (18), (19), and (20) are binary variables. $y_{c}$ represents whether the controller is placed on switch *c*, $x_{nc}$ represents whether switch *n* is within the control range of controller *c*, and $z_{e_{ij}}$ represents whether the control path $e_{ij}$ is in the set of shortest control paths $P_{ij}$.

(18) $$y_{c}=\left\{\begin{array}{l} 0,if\;the\;controller\;is\;not\;placed\;on\;node\;c\\ 1,if\;the\;controller\;is\;placed\;on\;node\;c \end{array}\right.,\forall c\in N,$$

(19) $$x_{nc}=\left\{\begin{array}{l} 0,if\;switch\;n\;is\;managed\;by\;controller\;c\\ 1,if\;switch\;n\;is\;not\;managed\;by\;controller\;c \end{array}\right.,\forall n\in N,\forall c\in C,$$

(20) $$z_{e_{ij}}=\left\{\begin{array}{l} 0,if\;the\;link\;is\;not\;in\;the\;set\\ 1,if\;the\;link\;is\;in\;the\;set \end{array}\right.,\forall e_{ij}\in P_{ij},\forall n_{i},n_{j}\in N,$$

### 3.3. Problem Formulation

To address the issue of distributed controller placement, it is necessary to determine how many controllers should be deployed, where the controllers are located, and to which controller the switches are assigned. Therefore, in this section, this is divided into two subproblems: the network partition subproblem and the controller placement subproblem.

#### 3.3.1. Network Partition Subproblem

The purpose of the network-partitioning stage is to determine the number of controllers reasonably. For this purpose, we establish a network partition model to optimize network performance while minimizing network deployment costs. Based on the network analysis, delay, switch security, and load are the main factors that affect the number of controllers. Therefore, the formula for network partitioning is as follows:

(21) $$minimize\;C_{place}=\sum_{i}\left(\sum_{j}d_{ij}\times \lambda_{i}\times \varphi_{i}\right),$$

where $C_{place}$ represents the network deployment cost, $d_{ij}$ represents the length of the propagation path between switch *i* and switch *j*, $\lambda_{i}$ represents the average flow request volume of switch *i*, and $\varphi_{i}$ represents the failure cost of switch *i*.

#### 3.3.2. Controller Placement Subproblem

For the distributed controller placement problem, we establish four models: delay model, security model, load model, and link cost model. At the same time, according to the requirements of network operators, we combine four objectives into a single-objective problem as a new model for solving the controller placement problem. Since the evaluation criteria for reaching the optimal value of each objective are inconsistent, we normalized them, and the multi-objective based controller placement was calculated by the following formula:

(22) $$\begin{array}{c} minimize\;\alpha \cdot \frac{D-D_{min}}{D_{max}-D_{min}}+\beta \cdot \frac{S_{max}-S}{S_{max}-S_{min}}+\gamma \cdot \frac{L-L_{min}}{L_{max}-L_{min}}+\delta \cdot \frac{C-C_{min}}{C_{max}-C_{min}},\\ s.t.\;(14\text{—}20). \end{array}$$

In this formula, to address controller placement for different network scenarios, α, β, γ, and δ represent the weights of the delay model, security model, load model, and link cost model, respectively, which are set by the ISP to match multiple service requirements.

## 4. MODECP Approach

**Figure 1:**
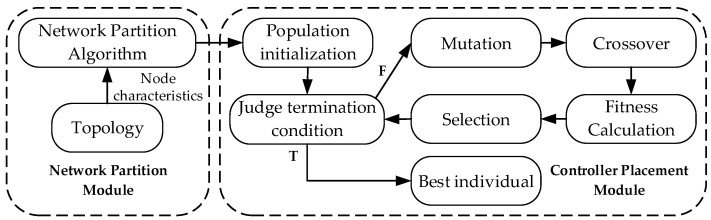
MODECP Approach.

In this section, to address this issue, we propose a heuristic-based multi-objective controller placement approach, MODECP, which is divided into the network-partitioning stage and the controller placement stage. Specifically, the network-partitioning stage aims to determine the number of controllers that should be placed reasonably by relying on the characteristics of the network through an improved affinity propagation algorithm. In the controller placement stage, we propose an improved multi-objective differential evolution algorithm to determine the placement position of the controllers in the network and the ownership relationship between the switches and the controllers. Based on the network partition stage and the controller placement stage, the MODECP approach can produce a distributed network management scheme that makes the network performance approximately optimal. Under different network requirements, various design objectives can be optimized, including minimizing network transmission delay, maximizing network security performance, minimizing controller load-balancing rate, and minimizing link overhead cost. In addition, for clarity, the research approach in this section is represented in Figure 1.

### 4.1. Network Partition Module

The purpose of the network partition module is to determine the number of network partitions according to the node characteristics of different network environments. In other words, in order to make various network performance indicators as good as possible, a reasonable number of controllers should be placed in the network. In this paper, the MODECP approach uses the affinity propagation algorithm to create a delay matrix, a security matrix, and a load matrix based on network characteristics.

The affinity propagation algorithm is a semi-supervised algorithm [41] that does not need to determine the number of clusters and the center of clusters in advance. The clustering foundation of the affinity propagation algorithm is the similarity between the network nodes. The preference *p* can be determined through the similarity matrix, and the number of clusters presents different results based on the value of preference *p*, which directly affects the selection of the number of clusters.

In order to obtain a reasonable number of controllers, which makes the network run for as long as possible without failure and congestion, we minimized the controller placement cost based on the improved affinity propagation algorithm, which includes network delay, nodes security, and load according to the model established in Section 3. The improved affinity propagation algorithm takes the negative value of the controller placement cost as its similarity matrix, as in Equation (23), where preference *p* selects the mean value of the similarity degree of each switch.

(23) X=−D×S×L,

Here, X is the similarity matrix, D is the reachable path delay matrix from each switch to other switches, S is the security matrix of the switch, and L is the load matrix of the switch.

The affinity propagation algorithm updates responsibility and availability through each iteration, which are denoted in Equations (24) and (25), respectively, and finally obtains a reasonable number of clusters. This pseudocode is described in Algorithm 1.

(24) $$r(i,k)\leftarrow x(i,k)-\underset{j\neq k}{max}\{x(i,j)+a(i,j)\},$$

(25) $$a(i,k)\leftarrow min\{0,r(k,k)+\sum_{k}max\{0,r(j,k)\}\},\;a(k,k)\leftarrow max\{0,r(j,k)\},$$

Here, a(i,j) is the availability of switch *j* to switch *i*, x(i,j) is the similarity between switch *j* and switch *i*, r(i,k) is the responsibility of switch *k* and switch *i*, a(i,k) is the availability of switch *k* to switch *i*, and r(k,k) is the responsibility of switch *k*.
**Algorithm 1** Network Partition Algorithm**Input**$G\left(N,E\right)$, Delay matrix, Security matrix, Load matrix, iteration *g***Output**The number of controllers *k*1Computing X and setting preference *p*2Initialize the responsibility matrix and the availability matrix3**for** *g*_0_*≤ g* or cluster center update **do**4  Iteratively update responsibility and availability according to Equations (24) and (25)5  *g*_0_ = *g*_0_ + 16**end for**7Calculate the number of cluster centers8**return** the number of cluster centers

### 4.2. Controller Placement Module

The purpose of the controller placement module is to select a certain number of switch nodes from all switches to deploy control nodes so that these nodes contain both control and forwarding functions. After that, the remaining switches are reasonably allocated to controllers located at different positions so that the network can operate normally with lower delay, higher security, more balanced load, and lower link overhead. The controller placement approach comprehensively considers factors such as network delay, node security, controller load balance, and link cost, and it searches for the approximate optimal candidate switch to place the controller. According to the different requirements of network operators, based on the formulation of the problem in Section 3, the controller placement can be transformed from a single-objective optimization problem to a multi-objective optimization problem. However, due to different optimization objectives, there is regularly mutual restriction. That is, when one objective is optimized, other objectives will not obtain the optimal result to a large extent. Therefore, there is regularly a Pareto-optimal solution set for multi-objective optimization problems. 

In this section, we solve the controller placement problem by normalizing the different objectives. We propose a heuristic-based improved multi-objective differential evolution algorithm to obtain an approximate Pareto-optimal solution set. Its main parts include: (1) Population initialization. (2) Population mutation. (3) Population crossover. (4) Fitness evaluation. (5) Optimal population selection.

In the improved multi-objective differential evolution algorithm, we believe that the position of the node where the controller is located and the ownership of the switch and controller are the main factors that constitute the chromosome. We obtain the main parameters of the construction algorithm from the input network topology information. The length of the chromosome is considered to be the total number of switches without controllers. The fitness function considers the factors of network delay, node security, controller load rate, and link overhead. A fitness evaluation function is constructed based on the model in Section 3 to select the optimal population according to the latitude and longitude geographic location, node connectivity, link betweenness centrality, and other characteristics. In addition, this approach also should set factors such as population size, mutation factor, and crossover factor. Additionally, the encoding method is natural number encoding. The normal operation of this algorithm is terminated by setting iteration times.

Firstly, a reasonable population initialization is performed. After setting the population number, the value of the chromosome is determined according to the decision space, while the number *k* is randomly selected as the controller location in the decision space. The range of the decision space is [0, *N* − 1]. The initialization of the population is the process of initializing both the controller position and the ownership of the switch and controller. The initialization occurred under the conditions of controller capacity constraint, switch–controller ownership constraint, and random initial population. The population initialization pseudocode is described in Algorithm 2.
**Algorithm 2** Initialization of the population**Input***k*, population size: *np*, chromosome length: *cl*, Ca, $\lambda_{i}$**Output**Initial population1Randomly generate *k* values in the range [0, *cl*−1] as *controller_set*2Initial *P*←null3**for** *i* < *np* **do**4  **for** *j* < *cl* **do**5    *P_i,j_*←randomly choose a value from *controller_set*6**end for**7  Calculate the load of each controller7  **if** the load of each controller exceeds capacity constraints **then**8    reproduce *P_i,j_*9**  end if**10**end for**11**return** Initial population and the set of controllers

Secondly, the difference operation is performed on the newly generated population. That is, two different vectors in the population are used to interfere with an existing vector so as to achieve mutation. We randomly select three distinct offspring vectors from the population to mutate based on a given mutation factor according to the formula $V_{i}^{g}=P_{r1}^{g}+F\cdot (P_{r2}^{g}-P_{r3}^{g});i=1,2,\ldots ,np$. In the mutation process, in order to ensure the validity of the selected controller, it is necessary to judge whether the controller assigned to each switch exists in the set of candidate controller positions previously selected in the mutant individual. In addition, the ownership of switches and controllers is also limited by the capacity of the controllers. If these conditions are not met, the mutated individuals are regenerated in the candidate controller set randomly. The pseudocode of the mutation process is described in Algorithm 3.
**Algorithm 3** Mutation**Input**Population, *controller_set*, Mutation parameter: F**Output**New population1Initial *Ps*←null2**for** *i* < *np* **do**3  randomly generate the numbers of the three offspring vectors,  where r1,r2,r3∈(1,np),r1≠r2≠r3
4  
$V_{i}^{g}=P_{r1}^{g}+F\cdot (P_{r2}^{g}-P_{r3}^{g});i=1,2,\ldots ,np$
5  **for** *j* < *cl* **do**6    **for** *c* < *controller_set* **do**7      **if** chromosome don’t in *controller_set* **then**8         randomly select a controller from *controller_set* as the newly        owning control node9**      end if**10**    end for**11**  end for**12**end for**13**return** mutate population

In order to enhance the diversity of the population, the new population is crossed to generate a variety of progeny vectors. For each individual in the population, the *g*-th generation population is crossed with its mutated new population based on the given crossover factor. By comparing with the crossover factor, the alleles of the new population are selected from the unmutated population and the mutant population. The pseudocode for the crossover process is described in Algorithm 4.
**Algorithm 4** Crossover**Input**Population, mutation population, crossover parameter: *cr***Output**New population1Initial *Ps*←null2Randomly generate *jrand* and a real number *r* of size (*np*, *cl*) between 0 and 1.3**for***i* < *np* **do**4  **for**
*j* < *cl* **do**5    **if** *r*(*i*,*j*) < *cr* or *j=jrand* **then**6      
$U_{i,j}^{g}$
←
$V_{i,j}^{g}$
7    
**else**
8      
$U_{i,j}^{g}$
←
$P_{i,j}^{g}$
9    
**end if**
10    
*Ps*
←
$U_{i,j}^{g}$
11  
**end for**
12**end for**13**return** crossover population

Finally, to select the optimal individual from the population, we utilize the greedy algorithm to select the population according to the evaluation result of the fitness function. The fitness function of each individual in the population is compared, and the individual with the best fitness function is selected and stored in the candidate optimal solution set. The best performance result in the candidate solution set is ultimately selected as the solution in the controller placement module, which effectively completes the selection of the controller position and the management of the switch. The pseudocode for the population selection process is described in Algorithm 5.
**Algorithm 5** Selection**Input**Population, crossover population**Output**the better solution1Initial *Ps*←null2Calculate the fitness of all individuals in the population3**if** $f(U_{i}^{g})$ < $f(P_{i}^{g})$ **then**4  
$Ps_{i}^{g}$
←
$U_{i}^{g}$
5**else**6  
$Ps_{i}^{g}$
←
$P_{i}^{g}$
7**end if**8**return** the better solution

In the differential evolution process, the evaluation of the fitness function is a critical step. The fitness of each individual distribution in each generation is calculated for the purpose of comparing the approximate optimal results. The pseudocode for the calculation process of the fitness function is described in Algorithm 6.
**Algorithm 6** Fitness Calculation**Input**Chromosome, *controller_set***Output**Fitness1Calculate *D*, *S*, *C* and *L* by Equations (5), (8), (10) and (13)2The fitness is calculated by normalizing to Equation (22)3**return** the fitness of chromosome

Through the above-mentioned operations of population initialization, mutation, crossover, selection, and fitness evaluation, the population can evolve repeatedly and cyclically. The algorithm stops running when the iteration reaches the maximum iteration times.

## 5. Simulation Results

In this section, we conduct extensive simulations of the proposed MODECP approach from multiple perspectives. In addition, we analyze the simulation results of the proposed algorithm and compare them with the traditional genetic algorithm and random placement algorithm.

### 5.1. Simulation Environment

*Experimental environment:* This experiment runs on an ordinary PC with an Intel Core i5-6600 CPU @3.30 GHz. All three algorithms are verified by Python 3.7.

*Network topology:* We evaluate the performance of the proposed approach on three topologies from the Internet Topology Zoo [42], namely the Xspedius topology with 34 nodes and 49 edges, the Bellcanada topology with 48 nodes and 65 edges, and the Uninett2010 topology with 74 nodes and 101 edges. Assuming that the request volume of each switch is a randomly generated value in [150, 250] kilorequests/s, the capacity of the controller is 150% of the average request volume of the switch, and the bandwidth of each link is 2 Gbps. The packet size for synchronization inter-controller is 200 bytes, and the synchronization interval is 5 s. We set packet_in packet size to 98 bytes, set packet_out packet size to 104 bytes, and ignore the payload in the packet [13]. We set the evaluation parameters, which are network delay, node security, load rate, and link cost.

The proposed algorithm is a differential evolution algorithm based on multiple objectives. Both the differential evolution algorithm and genetic algorithm are stochastic intelligent optimization algorithms. However, their parameters are different in crossover, mutation, and selection stages, which leads to the difference in accuracy and operation time between the two algorithms. In addition, the random placement algorithm is uncertain, but it considers the principle of minimizing delay for controller deployment. Therefore, we choose the genetic algorithm based on multiple objectives and the random placement algorithm based on minimum delay as the benchmark algorithms to prove the efficiency and effectiveness of the proposed algorithm, respectively.

*Proposed Algorithm*: In order to evaluate its validity and efficiency, the fitness of each population is calculated based on the multi-objective functions formed by fitting the network delay, network security, load rate, and link cost. Among them, we set the population size to 250, the number of iterations to 200, the mutation factor to 0.8, and the crossover factor to 0.8.

*Benchmark Algorithms:*

- Genetic Algorithm: To ensure the results’ efficiency, the genetic algorithm evaluates fitness based on the same multiple objectives as the proposed algorithm. Genetic algorithms search optimization occurred through continuous iteration of selection, crossover, and mutation. In this comparative experiment, the population size of the genetic algorithm is set to 250, the number of iterations is 200, the mutation factor is 0.8, and the crossover factor is 0.8.
- Random placement algorithm: To ensure the results’ validity, the algorithm randomly generates controller positions and allocates nearby switches according to the principle of minimum delay. The algorithm maintains great randomness and instability without iteration and optimization while the controller positions are selected.

In order to clearly compare the proposed approach with benchmark algorithms, the parameter settings of the three algorithms are shown in Table 3.

### 5.2. Simulation Results

#### 5.2.1. Performance Comparisons of Different Objective Models

We evaluate the algorithm performance of the improved differential evolution algorithm in single-objective and multi-objective situations and verify its effectiveness in four aspects, which are delay, security, load rate, and link overhead. To this end, we choose Xspedius, Bellcanada, and Uninett2010 topologies to conduct many simulation experiments to obtain the performance of MODECP.

**Figure 2:**
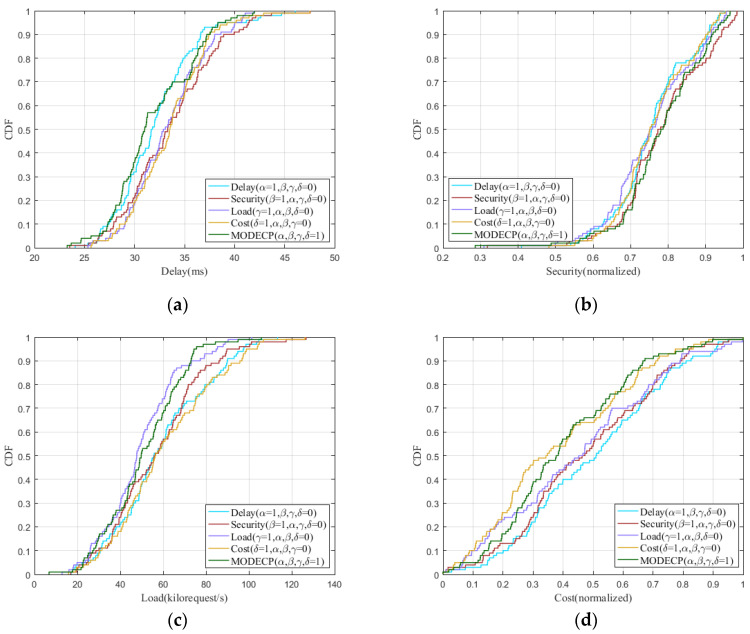
Cumulative distribution functions for different target states. (**a**) Performance of different states regarding end-to-end latency. (**b**) The performance of different states regarding node security. (**c**) The performance of different states regarding the control node load rate. (**d**) The performance of different states regarding the link overhead.

Figure 2 shows the cumulative distribution functions for multi-objective and different single-objective states. Through a large number of simulation experiments on multiple network topologies, the performance of the MODECP can be obtained under the single-objective conditions and simultaneous fitting of multi-objective conditions. Figure 2a shows that when the end-to-end delay is used as the evaluation parameter, the improved differential evolution algorithm considering only the delay performs the best compared with other objective situations. Since the MODECP considers four objectives at the same time, the end-to-end delay performance is inferior to that of the single-objective algorithm that only considers the delay, but it is better than that of the other three single-objective states. According to the security model in Section 3, security is inversely proportional to the probability of data loss. In other words, when links or nodes fail, the higher the probability of data loss is, the worse the network security performance is. Therefore, we hope for controllers to be deployed on nodes with a low probability of failure and control paths to be distributed on links with a low probability of failure. At this point, the larger the normalized value, the better the security of the network, according to Equation (8). Figure 2b–d shows the performance under five situations when node security, load rate, and link overhead, respectively, are used as evaluation parameters. Similarly, these experimental results show that the performance of the MODECP on three evaluation parameters is inferior to, or similar to, the case where only a single objective is considered.

To evaluate the impact of link failures on deployment results, we train the SVM model and predict the link state. We randomly generate the link traffic distribution and classify the link state as 0 or 1 to be used in the dataset for training the SVM model. We also simulate link traffic for five time periods in order to highlight the different link failure states due to traffic changes at different periods. The SVM model predicts the links that fail and tests the performance of MODECP in terms of delay, load, and link cost after link failures. Specifically, we vary the percentage of failed links in the network to evaluate performance in the simulation experiments, including 5%, 10%, and 15%.

**Figure 3:**
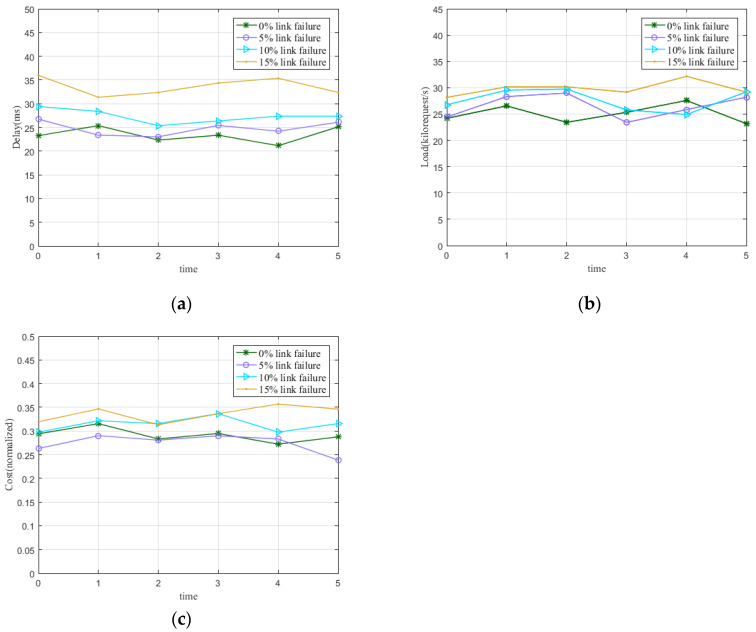
Performance evaluation of different time periods after 0%, 5%, 10%, and 15% link failures in Bellcanada topology. (**a**) Performance of different link failures in terms of delay. (**b**) Performance of different link failures in terms of load rate. (**c**) Performance of different link failures in terms of link overhead.

Figure 3 presents the performance trends in delay, load, and link overhead at various time periods after MODECP re-deployed the controller when changing the number of links that failed in the network. Based on comparing the number of failed links at 0%, 5%, 10%, and 15% in the Bellcanada topology, there is no significant difference in performance between no links failed and 5% of links failed. Due to the increased number of failed links, the control path length must be increased to deploy controllers with distribution switches to enable communication between the switches and controllers. As a result, the performance of the state with 15% link failures is significantly worse than the other states.

#### 5.2.2. Performance Comparisons of Different Algorithms 

For the same network topology, we evaluate the performance of the proposed algorithm with the same multi-objective based genetic algorithm and single-objective based random placement algorithm in terms of delay, security, load rate, and link overhead. To this end, we choose Bellcanada topology to conduct simulation experiments and obtain the performance of MODECP compared with other algorithms. Additionally, we set two delay states to describe the impact on the placement results by varying the delay calculation parameters. State 1 indicates that all delays are considered, namely the switch–controller propagation delay, the inter-controller propagation delay, transmission delay, and processing delay. State 2 indicates that only the switch–controller propagation delay and the inter-controller propagation delay are considered, without considering the transmission delay and processing delay. At the same time, we evaluate the time consumption for different algorithms and different topologies.

**Figure 4:**
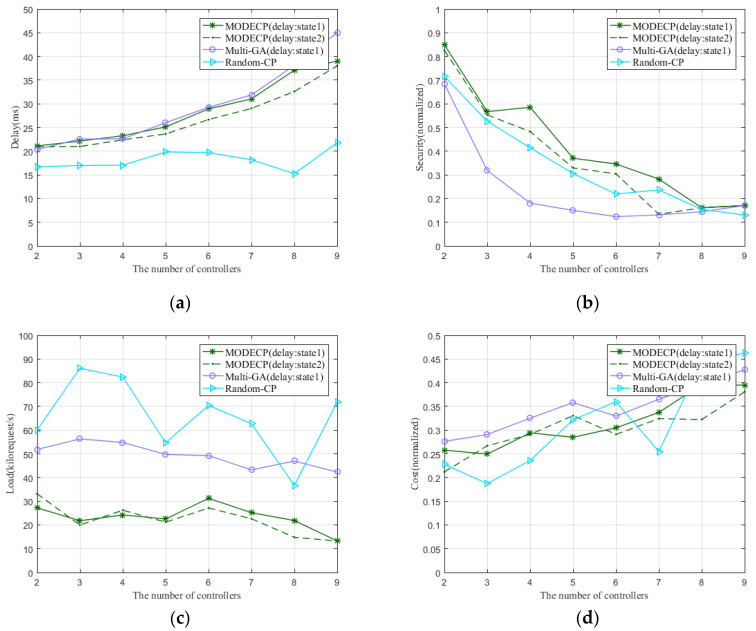
The performance of different algorithms under the Bellcanada topology. (**a**) Performance regarding end-to-end delay; (**b**) performance regarding node security; (**c**) performance regarding controlling node load rate; (**d**) performance regarding link cost.

Figure 4 shows the performance comparison of the MODECP with genetic algorithm and random placement algorithm under the same topology. For Bellcanada topology, the three algorithms have different performances in the evaluation parameters of end-to-end delay, security, load rate, and link overhead. No matter the point of view, MODECP in delay state 1 generally outperforms the same multi-objective based genetic algorithm in delay state 1 and the single-objective based random placement algorithm. Due to the uncertainty of the random placement algorithm, its performance exhibits a trend of unstable fluctuations. The performance trend of the MODECP and the genetic algorithm is basically similar, but the accuracy of the MODECP is better. In addition, by varying the delay model calculation parameters, it can be demonstrated that MODECP in state 2 performs slightly better than state 1 in terms of delay evaluation. The reason for this performance is that state 2 does not take processing delay and transmission delay into account as much as state 1. It can also be seen from the comparison that the difference in performance between state 1 and state 2 is minimal, which indicates that processing delay and transmission delay have less impact on the network performance. In terms of security, load, and link overhead, there is no significant trend of changing the delay model parameters in the deployment results.

**Figure 5:**
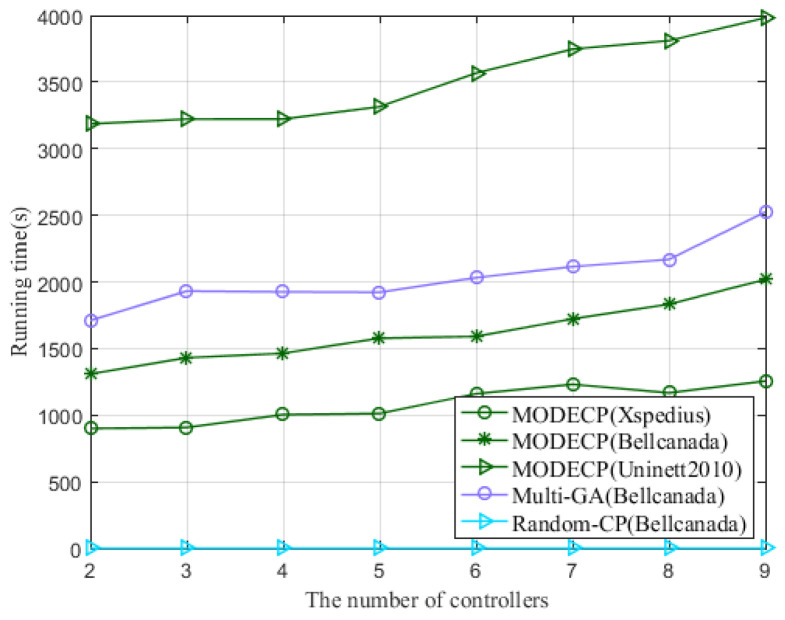
The running time of the algorithm.

Figure 5 shows the running time of the three algorithms under the Bellcanada topology and the running time of the proposed algorithm under different topologies, including Xspedius, Bellcanada, and Uninett2010. As can be seen from the description, the running time of the MODECP increases with the number of controllers for the same topology. For different topologies, the greater the number of switches in the network, the greater the range of switches that can be selected to place control nodes, so the longer the running time. Comparing the MODECP with the genetic algorithm and random placement algorithm, the running time of the random placement algorithm is consistently the shortest. This is because the random placement algorithm is based on the principle of minimum delay, and it does not have an iterative process to select the optimal result. Under the same conditions, the proposed algorithm has shorter running time and better convergence performance than the multi-objective based genetic algorithm. Since the mutated individuals in MODECP are obtained by differentiating the parent individuals and crossover with the parent individuals to generate new individuals, they are finally selected directly with the parent individuals. Therefore, the approximation effect of MODECP is better than that of the genetic algorithm.

#### 5.2.3. Performance Comparisons on Different Topologies 

Furthermore, to verify the universality of the MODECP, we evaluate the algorithm in three different topologies to obtain efficient solutions for distributed controller placement.

**Figure 6:**
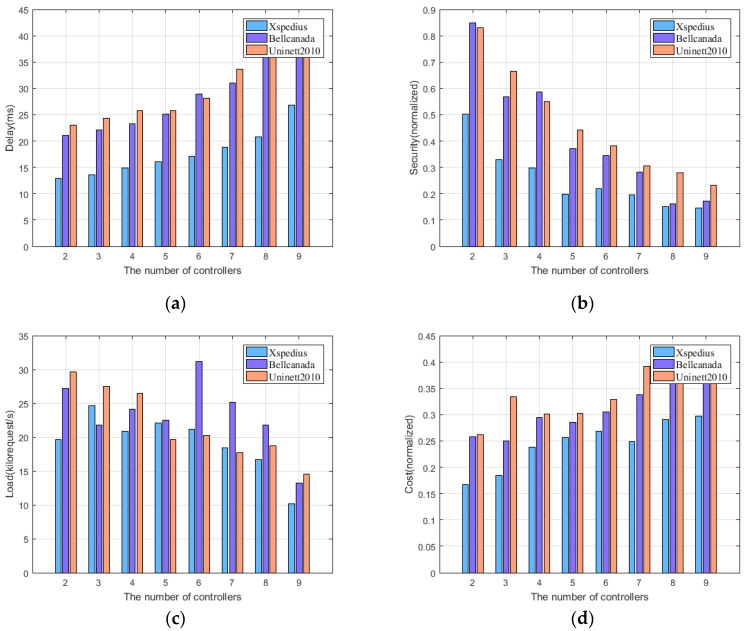
The performance of the proposed algorithm under different topologies. (**a**) performance regarding end-to-end delay; (**b**) performance regarding node security; (**c**) performance regarding controlling node load rate; (**d**) performance regarding link cost.

Figure 6 shows the performance of the proposed algorithm in three topological situations: Xspedius, Bellcanada, and Uninett2010. From Figure 6a, it can be seen that the performance of the proposed algorithm generally shows an increasing trend with the increase in the number of controllers regarding the end-to-end delay. When the number of controllers increases, the control scope of the controllers is reduced, resulting in a reduction in the switch–controller transmission delay, but the synchronization delay of the inter-controller will increase. Since the data packets synchronized by the controller are usually larger than the data packets transmitted by the switch controller, the performance of controller synchronization delay is more obvious. At the same time, the greater the number of nodes in the network, the greater the end-to-end delay. Figure 6b shows that node security generally shows a downward trend. When the number of controllers increases, the probability of the controllers being placed on switches with greater degree centrality will increase, so the security of the network decreases. That is, the more controllers deployed in the network, the worse the security performance. At the same time, when the number of nodes in the network is small, the search space for placing the controller becomes smaller, resulting in lower security. Therefore, the number of controllers deployed should not be too high to improve security. As can be seen from Figure 6c, the difference in topology has little effect on the load rate. Although the time interval of controller synchronization remains the same, the number of synchronizations between controllers will rise as the number of controllers grows. The link cost caused by synchronization increases, so Figure 6d shows that the link cost generally shows an increasing trend.

## 6. Conclusions

In this paper, we propose a heuristic-based MODECP approach to solve the distributed controller placement problem in SDN, including the network partition stage and the controller deployment stage. The appropriate number of controllers for placement is obtained based on the improved affinity propagation algorithm. Based on the improved multi-objective differential evolution algorithm, the placement position of the controller and the mapping relationship of the switches and controllers are obtained. The MODECP approach balances multiple optimization objectives to adapt to the different requirements of network operators, including network delay, switch security, controller load, and link overhead. Finally, extensive simulation results show that the MODECP approach exhibits better performance in terms of delay, security, controller load, and link cost, while reducing the running time. 

The placement of distributed controllers significantly affects network performance. Deep learning algorithms can be exploited to address this issue in future work to better optimize multiple objectives. At the same time, in addition to the influencing factors involved in this paper, exploring the elastic placement approach will also be a future research direction. Furthermore, the placement of controllers or servers in other application areas is also worth exploring. In virtual SDN networks (vSDNs), the placement of a network virtualization hypervisor also affects vSDNs’ performance [43]. Therefore, the placement algorithm can be used to address vSDN scalability issues and optimize network performance. In a content delivery network (CDN), it is necessary to deploy multiple caching servers in areas that users frequently access. Therefore, the placement algorithm can be used to solve the placement problem of content cache servers to improve network quality of service.

## Figures and Tables

**Table 1:** Summary of relevant studies.

Reference	Objectives	Number of Controllers	Approach
Reference [17]	the maximum propagation delay between the switches	×	hierarchical K-means algorithm
	the load of controllers		
Reference (CCPP) [18]	the maximum propagation delay between the switches	×	the capacitated K-center problem
	the capacity of controllers		
Reference (LBCPP) [19]	the load of controllers	×	based on topological potential and minimum cost flow
	the average delay between switches and controllers		
Reference [20]	the delay between switches and controllers	√	bargaining game
	the communication overhead inter-controller		
	the load of controllers		
Reference (RCP-DCP and RCP-DCR) [21]	single-link and node failures	×	Mixed Integer Linear Programming (MILP)
Reference (GreCo) [22]	the path delays and load of controllers	×	Binary Integer Program (BIP)
	the energy consumption		
Reference [23]	multiple-link failures	×	Betweenness Centrality Principle (BCP), Statistical Model (SM),
	worst-case delays		Minimizing Maximum Regret (MMR), Hurwicz Criterion (HC)
Reference [24]	single-link and multi-link failures	×	the heuristic algorithm and the greedy algorithm based on the Monte Carlo Simulation
Reference [26]	the delay between switches and controllers	√	hierarchical clustering
	the load of controllers		
Reference (CPCNS and CPSLF) [27]	node failures and link failures	×	a network states-traversal-based algorithm and greedy-based algorithm
Reference [28]	inter-controller delay, load distribution	√	genetic algorithm
Reference (CNCP) [29]	worst-case latency	×	simulated annealing heuristic
	multiple controller failures		
Reference (DDCP) [30]—Dynamic	the delay between switches and controllers	√	Deep Q-Network
	the Control Load (CL)		
	the Intra-Cluster Delay (ICD)		
	the Intra-Cluster Throughput (ICT)		
Reference (D4CPP) [31]—Dynamic	delay and load in the network	×	Deep Q-Network
Reference [32]—Dynamic	controllers–switches delay	×	a quadratic program
	inter-controllers delay		
	controllers load		

√ means the reference considers the number of controllers, × means the reference does not consider the number of controllers.

**Table 2:** Summary of symbols.

Notation	Description
$G\left(N,E\right)$	$\mathrm{The}\;\mathrm{network}\;\mathrm{with}\;\left|N\right|$$\;\mathrm{switches}\;\mathrm{and}\;\left|E\right|$ physical links.
$C=\{c_{1},c_{2},\ldots ,c_{k}\}$	The set of controllers, where *k* is the number of controllers.
$\lambda_{i}$	The average flow requests generated by switch i∈N.
Ca	The capacity of the controller.
$p_{sync}$	The frequency of synchronization between controllers.
$d_{ij}^{nc}$	The shortest transmission path length between switch *i* and controller *j*.
$d_{ij}^{cc}$	The shortest transmission path length between controller *i* and controller *j*.
v	The propagation rate of electrical signals in cables.
$\sigma_{c}$ $,\;\sigma_{n}$	The degree centrality of controller *c* and switch *n*, respectively.
$\sigma_{ij}$ $,\;\sigma_{ij}(e)$	The total number of shortest paths and the number of shortest paths through link *e* from switch *i* to switch *j*, respectively.
$l_{c}$ $,\;l_{opt}$	The actual load and the ideal load of the controller *c*.
$\rho_{ne}$ $,\;\rho_{ce}$	Link consumption for switch–controller communication and link consumption for inter-controller communication.
$A_{c}$	The set of switches managed by controller *c.*
$P_{ij}$	The set of control paths from switch *i* to switch *j.*
$B_{e}$	The bandwidth of link *e.*
$y_{j}$	Binary variable whose value is 1 if switch *j* has a controller placed and 0 otherwise.
$x_{ij}$	Binary variable whose value is 1 if switch *i* is linked to controller *j* and 0 otherwise.
$z_{e_{ij}}$	$\mathrm{Binary}\;\mathrm{variable}\;\mathrm{with}\;\mathrm{value}\;1\;\mathrm{if}\;\mathrm{link}\;e\mathrm{is}\;\mathrm{in}\;P_{ij}$, 0 otherwise.

**Table 3:** The Parameter Settings of algorithms.

Algorithm	Delay	Security	Load	Link Cost
Proposed Algorithm	√	√	√	√
Genetic Algorithm	√	√	√	√
Random Placement Algorithm	√	-	-	-

## Data Availability

Not applicable.
